# *TSC2* rare germline variants in non-tuberous sclerosis patients with neuroendocrine neoplasias

**DOI:** 10.1530/ERC-17-0286

**Published:** 2017-11-22

**Authors:** Paula Fontes Asprino, Rudinei Diogo Marques Linck, Jônatas Cesar, Florêncio Porto Freitas, Fernanda Christtanini Koyama, Rachel Simões Pimenta Riechelmann, Frederico Perego Costa, Paulo Marcelo Gehm Hoff, Pedro Alexandre Favoretto Galante, Diogo Meyer, Anamaria Aranha Camargo, Jorge Sabbaga

**Affiliations:** 1 Hospital Sírio-Libanês (HSL)São Paulo, São Paulo, Brazil; 2 Instituto do Câncer do Estado de São Paulo (ICESP)São Paulo, São Paulo, Brazil; 3 Instituto de Biociências – Universidade de São Paulo (IB-USP)São Paulo, São Paulo, Brazil

## Dear Editor,

Neuroendocrine neoplasias (NENs) represent a heterogeneous group of diseases with a wide spectrum of morbidity and lethality. These tumors can arise in almost any organ of the body, with the pancreas and the gastrointestinal tract being the most common primary sites (Yao *et al*. 2017). NENs are mainly considered sporadic diseases, although 5–20% of all cases occur in the context of genetic syndromes such as multiple endocrine neoplasia (MEN) type 1, MEN type 2, von Hippel–Lindau (VHL) disease, neurofibromatosis syndrome and tuberous sclerosis complex (TSC), which are caused by the presence of germline mutations in the *MEN1*, *RET*, *VHL*, *NF1* and *TSC1/TSC2* genes, respectively (Kim & Hong 2016). The incidence of NEN among patients with these syndromes varies considerably. Indeed, the incidence is very high in patients with MEN type 1 and is represented by isolated reports describing a small number of patients with TSC (Larson *et al*. 2012, Koc *et al*. 2017).

Notably, epidemiological studies have demonstrated that non-syndromic individuals with an affected relative are at an increased risk of developing gastroenteropancreatic (GEP) NENs. In individuals with affected siblings, the relative risk of GEP NEN increases by 13.4-fold, and an increased risk (2.3-fold) is present even in third-degree relatives (Neklason *et al*. 2016). Collectively, these epidemiological studies suggest the existence of additional genetic determinants leading to the development of GEP NENs in non-syndromic patients.

Here, we screened non-syndromic patients with GEP NEN for the presence of rare germline variants in genes previously associated with NEN-predisposing syndromes and searched for variants that could increase the risk of developing NENs without being associated with the corresponding syndromes.

Ninety-three patients with any grade GEP NENs and with no clinical or molecular diagnosis of MEN type 1 or 2, von-Hippel–Lindau disease, neurofibromatosis syndrome or TSC who were being treated at Hospital Sírio-Libanês or at Instituto do Câncer do Estado de São Paulo were enrolled in this study. All patients provided written informed consent, and the study was approved by the ethics committees of both institutions (HSL2015-15 and NP762/15).

Germline DNA was obtained from peripheral leucocytes. DNA libraries covering the coding regions of the *MEN1*, *RET*, *VHL*, *NF1*, *TSC1* and *TSC2* genes were prepared using TruSeq Custom Amplicon (Illumina) and sequenced on MiSeq (Illumina). Sequences were aligned to the human genome reference (hg19) using BWA, and nucleotide variants (SNVs and indels) were called using GATK. Only variants with predicted damaging impact on protein function (determined by SIFT or PolyPhen-2) and minor allele frequency (MAF) reported by ExAC (Exome Aggregation Consortium) lower than 0.1% were kept for further analysis.

We identified 8 patients with missense rare germline variants in *TSC2* (8.6%) and one in *RET* (1.1%). The presence of these variants was further validated by Sanger sequencing. There were no duplicated variants and no patient had more than one alteration. All missense rare germline variants identified in the *TSC2* and *RET* genes have a predicted damaging effect on protein function, but none is considered diagnostic for TSC or MEN2 syndromes by consensus criteria (Northrup & Krueger 2013) and, as of this writing, their clinical significance has not been established. Four of these variants have been described in LOVD database (TSC2_03292, TSC2_02023, TSC2_01014 and TSC2_0008), but none of them has been considered causative of the disease (Table 1).

**Table 1:** Variants identified by next-generation sequencing of 6 genes surveyed in 93 gastroenteropancreatic neuroendocrine neoplasia patients.

Patient # ID	11	51	10	54	64	16	81	39	75
Gene	*TSC2*	*TSC2*	*TSC2*	*TSC2*	*TSC2*	*TSC2*	*TSC2*	*TSC2*	*RET*
Genome change	chr16:g.2105420T>C	chr16:g.2138318C>T	chr16:g.2112532C>T	chr16:g.2127681G>A	chr16:g.2129637C>T	chr16:g.2121844C>T	chr16:g.2130325A>G	chr16:g.2120559G>A	chr10:g.43622171G>A
AA change	p.W167R	p.R1751C	p.A431V	p.A974T	p.R1122C	p.P669L	p.Y1186C	p.A607T	Splice site
Exon	6	41	13	26	29	19	30	17	19
dbSNP		rs781630603	rs202187148	rs556778889	rs397514938	rs794727221	rs137854421	rs45517203	
1000G total				0.0002			0.0002	0.0002	
ExAC total		0.00002511	0.0003556		0.00007914		0.000168	0.0006508	
ExAC European		0.00003065	0.0005986		0.00002428		0.00006161	0.0009623	
ESP6500si ALL							0.0002	0.0006	
SIFT score	0.002	0	0.004	0.006	0.011	0.005	0.014	0.155	0.008
PolyPhen2 score	0.999	1	1	0.999	0.003	0	0.999	0.999	1
LOVD ID	Not reported	Not reported	TSC2_03292	Not reported	TSC2_02023	Not reported	TSC2_01014	TSC2_00008	Not reported
ClinVar SIG	Not provided	Uncertain significance	Uncertain significance	Not provided	Not provided	Uncertain significance	Likely benign	Likely benign	Not provided
Interpro domain	Armadillo-like helical domain	Rap GTPase activating protein domain	Armadillo-like helical domain			Tuberin-type domain		Tuberin-type domain	

*TSC2* transcript: ENSbib219476.3. *RET* transcript: ENSbib355710.3. 1000G: values refer to minor allele frequency (MAF) reported by the total database. ExAC: MAF according to the total or European populations of this database. ESP6500si: MAF according to the total of this data set. Functional prediction of variants analyzed by SIFT and PolyPhen2. SIFT score: Deleterious (≤0.05); tolerated, (>0.05). PolyPhen2 score: Probably damaging (≥0.957), P: possibly damaging (0.453 ≤ pp2 ≤ 0.956); B: benign (pp2 ≤ 0.452). LOVD: whether variation has been described in LOVD database. Interpro domain describes if variant occurs at a conserved protein domain.

Patients with *TSC2* variants were carefully examined by clinical oncologists. Besides physical examination, imaging examinations were reassessed to identify the presence of any diagnostic features of TSC (Northrup & Krueger 2013). Magnetic resonance or computed tomography was used for abdominal/pelvic evaluation and renal ultrasonography was also used for specific renal evaluation. Thoracic evaluation was carried out using computed tomography, and echocardiography was used to evaluate the presence of cardiac rhabdomyoma. Unfortunately, fundoscopy examinations were not available and therefore the presence of ‘multiple retinal hamartomas’ or ‘retinal achromic patch’ was not actively evaluated. Three patients had no cranial imaging examinations and therefore the presence of ‘subependymal nodules’ or ‘subependymal giant cell astrocytoma’ were also not evaluated. Two patients had no abdominal images and one had no thoracic images. Importantly, no patient had symptoms related to TSC and, with the exception of two patients with more than one renal cyst, no other signs related to TSC were found among these patients.

To verify that the frequency of the identified deleterious rare germline variants in *TSC2* and *RET* was higher in our case series than the general population, we used two approaches. First, we queried the European non-Finnish population of the ExAC database, that we considered the most similar to our sample, to identify the number of individuals carrying deleterious variants (deleterious according to SIFT or probably damaging according to PolyPhen2), using the same criteria used in our case series. Because the deleterious variants of interest are rare, the probability of homozygosity or of an individual carrying multiple variants within a gene is exceedingly low. We therefore used the frequency of the variants within ExAC to predict the number of carriers in the sample. Conservatively, the number of alleles was inferred by considering the least sampled site of the gene. To estimate the total number of (diploid) individuals in the dataset, the number of alleles obtained was divided by two. The values derived are described on the contingency table for statistical testing (Table 2).

**Table 2:** Number of gastroenteropancreatic (GEP) neuroendocrine neoplasia (NEN) patients with and without missense variants in the *TSC2* and *RET* genes compared to unaffected individuals from the ExAC database.

	***TSC2* missense variants**				***RET* missense variants**			
	Carriers	Non-carriers	Odds ratio (*P*value)	CI (95%)	Carriers	Non-carriers	Odds ratio (*P*value)	CI (95%)
GEP NEN group	8	85			1	92		
ExAC (European non-Finnish)	107	3958	OR = 3.5 (*P* = 0.004)	Lower: 1.42 Upper: 7.42	127	11,742	OR = 1 (*P* = 1)	Lower: 0.02 Upper: 5.84
ExAC (Latin American)	52	2480	OR = 4.48 (*P* = 0.001)	Lower: 1.78 Upper: 9.90	11	789	OR = 0.78 (*P* = 1)	Lower: 0.01 Upper: 5.48
ExAC (all populations)	340	9491	OR = 2.63 (*P* = 0.016)	Lower: 1.09 Upper: 5.47	145	10,833	OR = 0.81 (*P* = 1)	Lower: 0.02 Upper: 4.71

The European non-Finnish samples are considered the closest ancestry population to our series. Because the sample size of the ExAC database varies among genes and sites, we conservatively inferred the number of individuals with and without deleterious variants by multiplying the frequency of the variants of interest and the sample size of the least sampled site for each of the three partitions of the data: European non-Finnish, Latin American and unfiltered. For example, in the European ExAC dataset, the *TSC2* genomic region with the lowest sample size has 2N = 8130 alleles, corresponding to 4065 individuals. As there are 107 rare variants in this sample, we assumed they represent 107 individuals within the group. All analyses were statistically significant (Fisher’s exact test).

We note that the assumption that each individual in the ExAC sample carries a single variant maximizes the number of carriers in the contingency table (since aggregation of mutations in a single individual would reduce the number of carriers). By maximizing the number of carriers in the control dataset our analysis is made conservative, strengthening our finding of an enrichment of deleterious variants among patients.

For *TSC2*, we found a significant increase in the number of rare germline variants among GEP NEN patients compared to controls (OR = 3.5, *P* value = 0.004, Fisher’s exact test). For *RET*, there was no difference in the number of deleterious variants (OR = 1) (Table 2). In addition, we drew 1000 random sets of 93 individuals from the European populations of the 1000 Genomes dataset, recording the number of deleterious rare germline variants per set. For *TSC2*, the number of deleterious variants among patients was significantly higher than that in the resampled data (mean in controls 1.47; empirical *P* value = 0.001). For *RET*, we found a lower number of deleterious variants among patients than controls (mean in controls 3.69; *P* value = 0.91).

The proteins hamartin and tuberin, respectively, encoded by the *TSC1* and *TSC2* genes, form a complex that downregulates the mTOR pathway. Variants that impair their function enhance the activity of this signaling pathway. Driver somatic variants leading to mTOR activation are frequent in NENs (Chou *et al*. 2016, Scarpa *et al*. 2017). Nevertheless, some may indeed correspond to germline (inherited) variants. A recently published study that examined whole-genome sequencing data from 98 pancreatic well-differentiated NENs described 5 patients (5.1%) carrying damaging rare germline variants in *TSC2* (Scarpa *et al*. 2017), providing an independent study consistent with our findings.

The relationship between TSC and NEN was primarily established by a series of case reports and two small studies of TSC patients (Larson *et al*. 2012, Koc *et al*. 2017). These studies reported a relatively high prevalence of well-differentiated pancreatic NENs in syndromic TSC patients, diagnosed mostly with functioning disease at a young age. However, the presence of NENs is not currently considered part of the TSC clinical spectrum (Northrup & Krueger 2013). In addition to what has been reported in the literature, our case series of non-syndromic patients carrying *TSC2* missense variants includes patients with tumors in the gastrointestinal tract as well as non-functioning and poorly differentiated NENs. We also showed that *TSC2* missense variants are present in patients with single lesions, with no affected relatives and with an average age at diagnosis of 51 years. This pattern, which is not suggestive of a genetic predisposition, can be explained by a possible low penetrance, as well as by the occurrence of de novo mutations in the *TSC2* gene, which have previously been described in up to two-thirds of TSC patients (Roberts 2004). These individuals normally carry large deletions or nonsense/out-of-frame mutations in the *TSC1/TSC2* genes, which result in the production of truncated, non-functional proteins (Northrup & Krueger 2013). Interestingly, none of the variants reported in our study results in a truncated protein, and the single amino acid changes caused may result in an impaired but still functional protein (Rosset *et al*. 2017).

In summary, we have uncovered an enrichment of *TSC2* missense rare germline variants in non-syndromic GEP NEN patients. This finding supports the role of the mTOR pathway in NEN pathogenesis and strongly suggests a contribution to inherited cancer predisposition. Of note, these missense rare germline variants are not associated with a TSC phenotype, and carriers cannot be clinically distinguished among GEP NEN patients. Closer follow-up studies or familial genetic screening should be performed in the future as well as functional assays that can determine the contribution of these variants to the net activation of the mTOR or other relevant pro-tumorigenic pathways.

## Declaration of interest

The authors declare no conflict of interest that could be perceived as prejudicing the impartiality of this article.

## Funding

This research was supported by Fundação de Amparo à Pesquisa do Estado de São Paulo (FAPESP), grant number 2015/12535-1 and by Ludwig Institute for Cancer Research – São Paulo.

## References

[bib1] Chou W-C Lin P-H Yeh Y-C Shyr Y-M Fang W-L Wang S-E Liu C-Y Chang P M-H Chen M-H Hung Y-P 2016 Genes involved in angiogenesis and mTOR pathways are frequently mutated in Asian patients with pancreatic neuroendocrine tumors. International Journal of Biological Sciences 12 1523–1532. (10.7150/ijbs.16233) 27994516 PMC5166493

[bib2] Kim JY Hong S-M 2016 Recent updates on neuroendocrine tumors from the gastrointestinal and pancreatobiliary tracts. Archives of Pathology and Laboratory Medicine 140 437–48. (10.5858/arpa.2015-0314-RA) 27128301

[bib3] Koc G Sugimoto S Kuperman R Kammen BF Karakas SP 2017 Pancreatic tumors in children and young adults with tuberous sclerosis complex. Pediatric Radiology 47 39–45. (10.1007/s00247-016-3701-0) 27639993

[bib4] Larson AM Hedgire SS Deshpande V Stemmer-Rachamimov AO Harisinghani MG Ferrone CR Shah U Thiele EA 2012 Pancreatic neuroendocrine tumors in patients with tuberous sclerosis complex. Clinical Genetics 82 558–563. (10.1111/j.1399-0004.2011.01805.x) 22035404

[bib5] Neklason DW VanDerslice J Curtin K Cannon-Albright LA 2016 Evidence for a heritable contribution to neuroendocrine tumors of the small intestine. Endocrine-Related Cancer 23 93–100. (10.1530/ERC-15-0442) 26604321 PMC4684974

[bib6] Northrup H Krueger DA 2013 Tuberous sclerosis complex diagnostic criteria update: recommendations of the 2012 international tuberous sclerosis complex consensus conference. Pediatric Neurology 49 243–254. (10.1016/j.pediatrneurol.2013.08.001) 24053982 PMC4080684

[bib7] Roberts PS 2004 Somatic mosaicism is rare in unaffected parents of patients with sporadic tuberous sclerosis. Journal of Medical Genetics 41 e69–e69. (10.1136/jmg.2003.014126) 15121797 PMC1735761

[bib8] Rosset C Netto CBO Ashton-Prolla P 2017 TSC1 and TSC2 gene mutations and their implications for treatment in Tuberous Sclerosis Complex: a review. Genetics and Molecular Biology 40 69–79. (10.1590/1678-4685-gmb-2015-0321) 28222202 PMC5409767

[bib9] Scarpa A Chang DK Nones K Corbo V Patch AM Bailey P Lawlor RT Johns AL Miller DK Mafficini A 2017 Whole-genome landscape of pancreatic neuroendocrine tumours. Nature 543 65–71. (10.1038/nature21063) 28199314

[bib10] Yao JC Hassan M Phan A Dagohoy C Leary C Mares JE Abdalla EK Fleming JB Vauthey JN Rashid A 2017 One hundred years after ‘carcinoid’: epidemiology of and prognostic factors for neuroendocrine tumors in 35,825 cases in the United States. Journal of Clinical Oncology 26 3063–3072. (10.1200/JCO.2007.15.4377) 18565894

